# Partial Molar Volumes of 15-Crown-5 Ether in Mixtures of *N*,*N*-Dimethylformamide with Water

**DOI:** 10.1007/s10953-014-0138-7

**Published:** 2014-02-08

**Authors:** Magdalena Tyczyńska, Małgorzata Jóźwiak

**Affiliations:** Department of Physical Chemistry, University of Lodz, Pomorska 165, 90-236 Lodz, Poland

**Keywords:** *N,N*-Dimethylformamide–water mixture, 15-Crown-5, Density, Partial molar volume, Hydrophobic hydration

## Abstract

The density of 15-crown-5 ether (15C5) solutions in the mixtures of *N,N*-dimethylformamide (DMF) and water (H_2_O) was measured within the temperature range 293.15–308.15 K using an Anton Paar oscillatory U-tube densimeter. The results were used to calculate the apparent molar volumes (*V*_*Φ*_) of 15C5 in the mixtures of DMF + H_2_O over the whole concentration range. Using the apparent molar volumes and Redlich and Mayer equation, the standard partial molar volumes of 15-crown-5 were calculated at infinite dilution ($V_{\text{m}}^{^\circ }$). The limiting apparent molar expansibilities (*α*) were also calculated. The data are discussed from the point of view of the effect of concentration changes on interactions in solution.

## Introduction

Studies of the properties of solutions connected with their density within a wide range of concentration and temperatures are a valuable source of information. The volumetric functions and the temperature coefficient of expansion make it possible to know not only the physical properties of the solute and solvent under investigation, but they can also be used to verify changes occurring in the solution structure brought about by intermolecular interactions [1–7].

In our measurements solutions of 15C5 in DMF + H_2_O mixtures were used. The special properties of water can be changed under the influence of a substance dissolved in it. Depending on the hydrophobic–hydrophilic properties of the solute, the three-dimensional network of hydrogen bonds can be reinforced or weakened, which may show itself in changes in the density of aqueous solutions (molar volume) and changes in chemical potential [8].

The investigation of changes in the interactions among the components of mixed solvent and solute with hydrophobic–hydrophilic properties requires one to choose an organic solvent that does not specifically react with water. This will allow one to observe changes in the interactions among the mixture components and solute, brought about by the process of preferential solvation including the hydrophobic hydration of the solute. This requirement is met by *N,N*-dimethylformamide (DMF). Its molecules have groups with hydrophilic and hydrophobic properties that are almost mutually compensated, which makes them a convenient object for investigations in a mixture with water [9]. DMF is used as model substance for investigation the interactions of small biological molecules serving as a model compound for protein [10]. The amide group can serve as a model of the peptide bond, and interactions between hydroxyl and amide groups play an important role in the solvation of peptides in aqueous solutions [11].

Crown ethers constitute a very interesting class of compounds characterized by hydrophilic–hydrophobic properties resulting from their structure. Therefore they are capable of selective complexation of ions and small organic molecules; thus, for example, they are used to make ion selective electrodes [12].

One can find many studies more or less successfully describing the effect of crown ether molecules on the interactions in mixed aqueous–organic and organic–organic solvents. Such studies have been carried out by means of various experimental techniques [13–19]. In the literature, there are few papers on the volumetric properties of crown ethers in water or in mixtures of water with an organic solvent [16, 20].

In this paper we present the effect of the mixed solvent composition and temperature on the volumetric properties of the system 15C5 + DMF + H_2_O. The data are compared with analogous results previously obtained for the solutions of urea (a hydrophilic compound) in the mixtures of DMF and water [21].

## Experimental and Method

### Materials

15-crown-5 (Aldrich, w = 0.98) was used as received. DMF (Aldrich, mass fraction = 0.99) was purified and dried according to the procedures described in the literature [22, 23] and distilled under vacuum. Water content, determined by the Karl Fisher method, was lower than 0.01 %. To prepare aqueous solutions, triple-distilled and degassed water was used.

### Measurements

The densities of the 15C5 solutions within the whole concentration range of mixed solvent, at temperatures *T*/K = 293.15, 298.15, 303.15 and 308.15, were measured with the use of an Anton Paar densimeter, model DMA 5000 with an oscillatory U-tube, whose uncertainty of density and temperature measurements are ±5 × 10^−3^ kg·m^−3^ and ±0.01 K, respectively, and the temperature stability is ±0.005 K. The densimeter was calibrated with the use of pure water. The obtained value of water density equal to 997.046 kg·m^−3^ at a temperature of 298.15 K is in agreement with the literature data [24]. The mixed solvent DMF + H_2_O and solutions of 15-crown-5 in DMF + H_2_O were prepared by weight using electronic balances with an accuracy of ±1 × 10^−2^ and ±1 × 10^−5^ g, respectively. The values of experimental densities of pure *N,N*-dimethylformamide are compared with literature data and collected in Table 1. The values of the solution densities obtained as a function of molality, *m* (*m* expressed as moles of 15-crown-5 per kilogram of solvent) at investigated temperatures, are presented in Table 2.

**Table 1:** Experimental density of *N,N*-dimethylformamide and literature data

Substance	*ρ* × 10^3^ (kg·m^−3^)			
	*T* = 293.15 K	*T* = 298.15 K	*T* = 303.15 K	*T* = 308.15 K
DMF	0.948737	0.943971	0.939196	0.934420
	0.94939^a^	0.94460^a^	0.93983^a^	0.93505^a^
	0.95045^b^	0.94559^b^	0.94069^b^	0.93561^b^
	0.948051^c^	0.942915^c^	0.938876^c^	0.933964^c^
	0.94917^d^	0.944^e^	0.9386^h^	0.9344^h^
		0.94381^f^		
		0.94403^g^		

^a^Berna-García et al. [25]

^b^Marchetti et al. [26]

^c^Sharlin et al. [27]

^d^Tôrres et al. [28]

^e^Bakshi et al. [29]

^f^Bendová et al. [30]

^g^Tong-Chun et al. [31]

^h^Akhtar et al. [32]

**Table 2:** Experimental densities, *ρ*, of 15-crown-5 in the DMF + H_2_O mixtures at temperatures *T* = (293.15, 298.15, 303.15 and 308.15) K

*x*_2_	*m* (mol·kg^−1^)	*ρ* × 10^3^ (kg·m^−3^)			
		*T* = 293.15 K	*T* = 298.15 K	*T* = 303.15 K	*T* = 308.15 K
0.00	0	0.948737	0.943971	0.939196	0.934420
	0.02548	0.949612	0.944847	0.940074	0.935300
	0.05941	0.950756	0.945992	0.941221	0.936447
	0.08622	0.951640	0.946877	0.942114	0.937343
	0.11692	0.952642	0.947884	0.943125	0.938359
	0.13341	0.953166	0.948402	0.943644	0.938876
	0.17510	0.954477	0.949715	0.944959	0.940197
	0.20937	0.955535	0.950789	0.946034	0.941275
	0.24408	0.956576	0.951832	0.947081	0.942326
0.10	0	0.953731	0.948879	0.944216	0.939439
	0.03367	0.954840	0.949990	0.945328	0.940554
	0.06351	0.955807	0.950958	0.946296	0.941523
	0.09159	0.956705	0.951856	0.947199	0.942428
	0.12396	0.957723	0.952879	0.948218	0.943453
	0.15189	0.958575	0.953736	0.949088	0.944310
	0.18502	0.959590	0.954751	0.950095	0.945334
	0.20868	0.960304	0.955451	0.950810	0.946049
	0.25199	0.961562	0.956728	0.952086	0.947332
0.20	0	0.959310	0.954573	0.949823	0.945045
	0.04255	0.960655	0.955919	0.951171	0.946398
	0.07195	0.961569	0.956833	0.952086	0.947314
	0.09842	0.962382	0.957646	0.952903	0.948136
	0.12857	0.963288	0.958561	0.953820	0.949060
	0.15401	0.964054	0.959321	0.954584	0.949819
	0.18505	0.964960	0.960245	0.955506	0.950752
	0.21101	0.965727	0.961002	0.956267	0.951517
	0.25734	0.967045	0.962328	0.957596	0.952839
0.30	0	0.965705	0.960992	0.956253	0.951493
	0.02471	0.966441	0.961730	0.956994	0.952236
	0.05485	0.967328	0.962619	0.957884	0.953130
	0.08073	0.968082	0.963374	0.958637	0.953883
	0.10921	0.968901	0.964195	0.959466	0.954715
	0.13379	0.969599	0.964897	0.960160	0.955410
	0.16111	0.970369	0.965665	0.960939	0.956192
	0.17732	0.970821	0.966119	0.961394	0.956647
	0.21842	0.971953	0.967254	0.962534	0.957787
0.40	0	0.972987	0.968296	0.963577	0.958796
	0.02690	0.973758	0.969067	0.964349	0.959571
	0.05893	0.974663	0.969974	0.965259	0.960484
	0.07837	0.975204	0.970517	0.965806	0.961031
	0.11204	0.976138	0.971452	0.966740	0.961967
	0.13346	0.976713	0.972038	0.967327	0.962556
	0.16708	0.977630	0.972948	0.968239	0.963471
	0.19964	0.978498	0.973818	0.969110	0.964344
	0.23028	0.979305	0.974627	0.969920	0.965157
0.50	0	0.980849	0.976200	0.971517	0.966802
	0.02234	0.981473	0.976823	0.972141	0.967426
	0.05427	0.982354	0.977704	0.973025	0.968309
	0.07937	0.983037	0.978387	0.973710	0.968992
	0.10775	0.983803	0.979151	0.974479	0.969758
	0.12484	0.984258	0.979608	0.974936	0.970217
	0.16227	0.985245	0.980597	0.975928	0.971207
	0.19826	0.986181	0.981530	0.976867	0.972146
	0.22877	0.986960	0.982314	0.977652	0.972930
0.60	0	0.988582	0.984016	0.979419	0.974785
	0.02030	0.989155	0.984587	0.979990	0.975354
	0.03362	0.989529	0.984960	0.980362	0.975726
	0.06490	0.990396	0.985826	0.981225	0.976588
	0.09303	0.991169	0.986597	0.981997	0.977360
	0.12367	0.992000	0.987427	0.982827	0.978187
	0.15369	0.992802	0.988226	0.983625	0.978984
	0.18395	0.993600	0.989023	0.984420	0.979780
	0.25543	0.995445	0.990865	0.986260	0.981619
0.70	0	0.995521	0.991167	0.986768	0.982319
	0.03136	0.996464	0.992101	0.987695	0.983239
	0.06435	0.997443	0.993070	0.988659	0.984194
	0.09125	0.998224	0.993844	0.989431	0.984956
	0.11435	0.998893	0.994508	0.990088	0.985616
	0.15157	0.999956	0.995565	0.991137	0.986646
	0.18098	1.000785	0.996387	0.991953	0.987424
	0.20196	1.001369	0.996967	0.992529	0.988026
	0.23981	1.002417	0.998008	0.993562	0.989070
0.80	0	0.999822	0.995938	0.991999	0.987998
	0.03182	1.000884	0.996986	0.993035	0.989021
	0.06761	1.002061	0.998150	0.994180	0.990158
	0.10137	1.003158	0.999234	0.995264	0.991212
	0.13026	1.004090	1.000146	0.996151	0.992096
	0.15750	1.004958	1.001001	0.996994	0.992930
	0.19474	1.006118	1.002143	0.998121	0.994039
	0.20937	1.006572	1.002591	0.998563	0.994478
	0.26742	1.008344	1.004339	1.000287	0.996181
0.90	0	0.999747	0.996896	0.993931	0.990857
	0.02425	1.000667	0.997792	0.994803	0.991718
	0.06077	1.002037	0.999125	0.996102	0.993000
	0.10132	1.003533	1.000583	0.997525	0.994401
	0.11740	1.004122	1.001160	0.998083	0.994952
	0.13681	1.004826	1.001846	0.998748	0.995613
	0.17294	1.006122	1.003106	0.999979	0.996827
	0.20232	1.007166	1.004121	1.000967	0.997804
	0.23732	1.008391	1.005317	1.002131	0.998952
0.92	0	0.999213	0.996655	0.993967	0.991148
	0.03136	1.000408	0.997816	0.995106	0.992268
	0.06265	1.001589	0.998962	0.996231	0.993374
	0.09774	1.002895	1.000233	0.997477	0.994599
	0.12799	1.004011	1.001315	0.998539	0.995644
	0.15895	1.005139	1.002411	0.999614	0.996700
	0.19205	1.006334	1.003571	1.000751	0.997819
	0.21136	1.007023	1.004239	1.001408	0.998464
	0.26014	1.008749	1.005912	1.003051	1.000078
0.94	0	0.998613	0.996383	0.993997	0.991465
	0.03717	1.000020	0.997759	0.995345	0.992788
	0.06953	1.001232	0.998944	0.996502	0.993923
	0.09583	1.002213	0.999904	0.997433	0.994832
	0.13217	1.003545	1.001207	0.998721	0.996094
	0.16474	1.004731	1.002368	0.999851	0.997212
	0.20262	1.006084	1.003694	1.001159	0.998491
	0.22400	1.006831	1.004423	1.001871	0.999187
	0.27371	1.008583	1.006135	1.003547	1.000829
0.96	0	0.998064	0.996186	0.994128	0.991899
	0.02961	0.999179	0.997277	0.995195	0.992944
	0.06577	1.000529	0.998597	0.996484	0.994206
	0.09853	1.001742	0.999775	0.997638	0.995337
	0.12369	1.002660	1.000674	0.998517	0.996199
	0.15178	1.003682	1.001671	0.999488	0.997147
	0.18196	1.004770	1.002731	1.000525	0.998162
	0.21265	1.005857	1.003794	1.001564	0.999177
	0.25315	1.007279	1.005182	1.002920	1.000502
0.98	0	0.997773	0.996269	0.994553	0.992644
	0.02909	0.998855	0.997327	0.995588	0.993659
	0.06203	1.000069	0.998512	0.996751	0.994798
	0.09171	1.001153	0.999570	0.997786	0.995811
	0.12209	1.002250	1.000647	0.998833	0.996846
	0.15501	1.003428	1.001796	0.999969	0.997943
	0.18354	1.004440	1.002784	1.000930	0.998902
	0.20860	1.005323	1.003649	1.001774	0.999726
	0.24953	1.006746	1.005039	1.003135	1.001057
1.00	0	0.998199	0.997043	0.995649	0.994041
	0.02470	0.999077	0.997898	0.996488	0.994859
	0.06100	1.000348	0.999145	0.997711	0.996065
	0.09177	1.001429	1.000189	0.998745	0.997078
	0.11536	1.002244	1.000985	0.999524	0.997845
	0.13029	1.002753	1.001488	1.000020	0.998329
	0.17530	1.004282	1.002977	1.001491	0.999777
	0.20216	1.005193	1.003866	1.002358	1.000628
	0.23731	1.006360	1.005023	1.003489	1.001745

*x*_2_ is the mole fraction of water in the mixture

## Results and Discussion

Using the density values of 15-crown-5 (15C5) in DMF + H_2_O mixtures (Table 2), the apparent molar volume of 15C5 in DMF + H_2_O mixtures was calculated using Eq. 1:

1 $$ V_{\varPhi } = M/\rho - 1000 \cdot (\rho - \rho_{\text{o}} )/m \cdot \rho \cdot \rho_{\text{o}} $$

where *M* is a molar mass of 15C5, *ρ*_o_ is the density of DMF + H_2_O mixtures, *ρ* indicates the density of the 15C5 + DMF + H_2_O system and *m* is the concentration of 15C5 in moles per kilogram of solvent. The dependence of *V*_*Φ*_ on the solute molality, *V*_*Φ*_ = *f*(*m*), can be described by Eq. 2:

2 $$ V_{\varPhi } = V_{\varPhi }^{^\circ } + b_{\text{V}} m $$

Equation 2 is a special case of the dependence proposed by Redlich and Meyer [33]:

3 $$ V_{\varPhi } = V_{\varPhi }^{^\circ } + S_{\text{V}} \sqrt m + b_{\text{V}} m $$

where *S*_V_ and *b*_V_ are empirical parameters. The parameter *S*_V_ is connected with the interaction of ions in solution, which can be calculated from Debye–Hückel’s limiting law. For solutions of nonelectrolytes, *S*_V_ = 0 [34]. The parameter *b*_V_ provides information about the character of interactions of solute molecules between themselves.

The values of *b*_V_ obtained as a function of the molar fraction of water are listed in Table 3. Considering the considerable standard deviations of the values of *b*_V_, these values will be discussed in qualitative terms. Up to *x*_2_ = 0.9 the values of *b*_V_ are positive. In the mixture with high water content, i.e. *x*_2_ > 0.9, these values are negative. The negative sign of coefficient *b*_V_ reflects the hydrophobic properties of 15C5 molecules [35, 36]. As can be seen the values of *b*_V_ decrease with increasing water content in the mixtures. This means that the interactions between the molecules of solute are becoming stronger [37, 38]. This is consistent with hydrophobic hydration of 15C5 molecules in aqueous medium and their enclosure in clathrate-like water structures.

**Table 3:** Coefficient *b*_V_ of Eq. 2

*x*_2_	*b*_V_ × 10^6^ (m^3^·kg·mol^−2^)			
	*T* = 293.15 K	*T* = 298.15 K	*T* = 303.15 K	*T* = 308.15 K
0.00	4.71 ± 0.10	4.48 ± 0.24	4.46 ± 0.22	4.32 ± 0.30
0.10	2.99 ± 0.18	3.04 ± 0.14	2.69 ± 0.13	2.71 ± 0.18
0.20	1.81 ± 0.15	1.43 ± 0.07	1.30 ± 0.07	1.34 ± 0.22
0.30	0.76 ± 0.03	0.82 ± 0.04	0.88 ± 0.23	1.06 ± 0.24
0.40	0.79 ± 0.18	0.56 ± 0.04	0.61 ± 0.09	0.75 ± 0.06
0.50	0.80 ± 0.05	0.59 ± 0.04	0.35 ± 0.08	0.47 ± 0.05
0.60	0.64 ± 0.06	0.52 ± 0.09	0.57 ± 0.11	0.36 ± 0.12
0.70	0.91 ± 0.14	0.62 ± 0.16	0.68 ± 0.10	0.85 ± 0.51
0.80	0.42 ± 0.12	0.68 ± 0.10	0.96 ± 0.28	1.06 ± 0.13
0.90	0.51 ± 0.04	0.41 ± 0.09	0.34 ± 0.08	0.27 ± 0.04
0.92	−0.25 ± 0.04	−0.17 ± 0.02	−0.19 ± 0.02	−0.19 ± 0.02
0.94	−0.42 ± 0.18	−0.51 ± 0.20	−0.72 ± 0.19	−0.68 ± 0.25
0.96	−0.97 ± 0.15	−0.60 ± 0.08	−0.55 ± 0.03	−0.38 ± 0.08
0.98	−0.90 ± 0.04	−0.96 ± 0.06	−0.86 ± 0.12	−0.92 ± 0.12
1.00	−1.71 ± 0.29	−1.65 ± 0.15	−1.59 ± 0.14	−1.44 ± 0.10

± is the standard deviation

For urea [21] the decreasing values of *V*_Φ_ with increasing urea content in the mixture testify to the existence of increasing short range attractions between urea molecules [37]. In the case of the DMF + H_2_O + urea system, the values of *b*_V_ are negative but they become less negative with increasing water content in the mixtures, which indicates hydrophilic properties of urea and that the solute–solute interactions have become weaker [21].

**Fig. 1:**
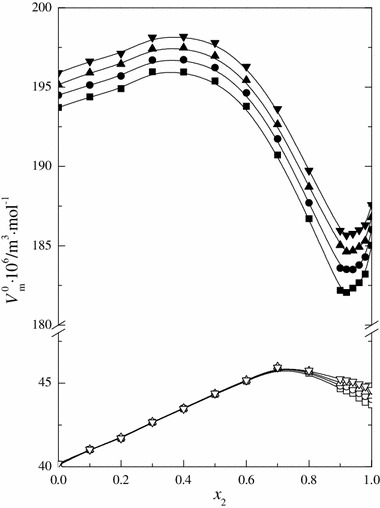
Standard partial molar volume ($V_{\text{m}}^{^\circ }$) of 15-crown-5 (*full symbol*) and urea [21] (*open symbol*) in the DMF + H_2_O mixtures for various temperatures: *filled square* 293.15 K, *filled circle* 298.15 K, *filled triangle* 303.15 K, *filled tilled triangle* 308.15 K as a function of the mole fraction of water

The values of the standard partial molar volume, $V_{\text{m}}^{^\circ }$ ($V_{\varPhi }^{^\circ }$ = $V_{\text{m}}^{^\circ }$), of 15C5 obtained by the extrapolation of *V*_*Φ*_ data are presented in Table 4 and in Fig. 1 as a function of the molar fraction of water. The same figure also shows the values of the standard partial molar volume of urea with hydrophilic properties within the same range of concentrations and temperatures that were published in our previous paper [21]. As is seen in Fig. 1, for 15C5 showing hydrophobic properties, the values of the standard partial molar volume in the mixture of DMF and water are considerably higher than those of urea. This difference also directly results from the considerable differences in the molecular sizes of the compounds. Analyzing the dependence $V_{\text{m}}^{^\circ } = f\left( {x_{2} } \right)$ for 15C5, we observe an increase in the partial molar volume with increasing water content in the mixtures up to *x*_2_ ≈ 0.4. This is probably due to the interactions between the polar groups of DMF and water. In a solution with low water content, the interactions between DMF and water molecules dominate. Some authors [11, 42–50] proposed the possibility of clathrate or complex formation (DMF∙(H_2_O)_*n*_, *n* = 1–4). This would be the reason that the molecules of 15C5 are mainly solvated by the organic co-solvent.

**Table 4:** Partial molar volume of 15-crown-5, $V_{\text{m}}^{^\circ }$, in the DMF + H_2_O mixtures

*x*_2_	$V_{\text{m}}^{^\circ }$ × 10^6^ (m^3^·mol^−1^)			
	*T* = 293.15 K	*T* = 298.15 K	*T* = 303.15 K	*T* = 308.15 K
0.00	193.72 ± 0.01	194.49 ± 0.04	195.16 ± 0.03	195.90 ± 0.04
0.10	194.39 ± 0.03	195.13 ± 0.02	195.90 ± 0.20	196.63 ± 0.09
0.20	194.90 ± 0.02	195.70 ± 0.01	196.45 ± 0.01	197.13 ± 0.04
0.30	195.97 ± 0.01	196.70 ± 0.01	197.42 ± 0.03	198.14 ± 0.03
0.40	195.96 ± 0.03	196.73 ± 0.01	197.47 ± 0.01	198.19 ± 0.01
0.50	195.39 ± 0.01	196.23 ± 0.01	196.97 ± 0.01	197.80 ± 0.01
0.60	193.79 ± 0.01	194.64 ± 0.01	195.44 ± 0.01	196.31 ± 0.02
0.70	190.72 ± 0.02	191.74 ± 0.02	192.65 ± 0.02	193.64 ± 0.08
0.80	186.70 ± 0.20	187.71 ± 0.02	188.71 ± 0.04	189.75 ± 0.02
0.90	182.18 ± 0.01	183.59 ± 0.01	185.02 ± 0.01	185.96 ± 0.01
0.92	182.05 ± 0.01	183.51 ± 0.01	184.62 ± 0.01	185.66 ± 0.01
0.94	182.33 ± 0.03	183.50 ± 0.03	184.68 ± 0.03	185.76 ± 0.04
0.96	182.67 ± 0.02	183.78 ± 0.01	184.91 ± 0.01	185.99 ± 0.01
0.98	183.21 ± 0.01	184.29 ± 0.01	185.30 ± 0.02	186.31 ± 0.02
1.00	185.02 ± 0.04	186.02 ± 0.02 186.4^a^ 186.2^b^ 186.06^c^ 186.46^d^	186.79 ± 0.02	187.59 ± 0.01

*x*_2_ is the mole fraction of water in the mixture

± is the standard deviation

^a^Dagade et al. [20]

^b^Bernal et al. [39]

^c^Høiland [40]

^d^Zielenkiewicz et al. [41]

**Fig. 2:**
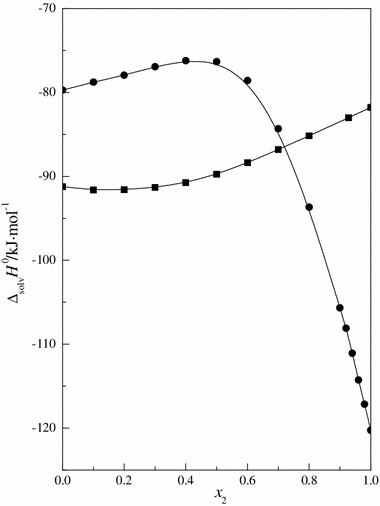
Standard solvation enthalpy (Δ_solv_ *H*°) (see the text) of: *filled circle*, 15-crown-5; and *filled square*, urea in the solvent mixture (DMF + H_2_O) at 298.15 K as a function of the mole fraction of water

The ordering of the mixed solvent structure and the presence of 15C5 molecules solvated by DMF most probably cause an increase in the values of $V_{\text{m}}^{^\circ }$ within the concentration range from *x*_2_ = 0 to 0.4. Further increase in the water content in the mixture probably causes the organic solvent molecules to be replaced by water molecules in the solvation shell. Taking into account the fact that the addition of water, which is a polar molecule, is a highly disadvantageous process, it may be expected that the system will tend to minimize the disadvantageous interactions between solute and water molecules. As a result of this, nonpolar molecules tend to interact among themselves, thereby reducing the number of contacts with water, which results in the superposition of solvation sheaths and the release of water molecules from the solvation shell into the bulk solvent [20]. The outcome of this phenomenon is the dissolution of 15C5 showing a considerable decrease in the value of $V_{\text{m}}^{^\circ }$ up to *x*_2_ ≈ 0.92. In a water-rich mixture, when *x*_2_ > 0.92, one can observe an increase in the standard partial molar volume of 15C5, which is directly caused by the phenomenon of hydrophobic hydration [39, 40]. Frank and Evans [51] have suggested that water forms cages around nonpolar solutes. The resultant structures are the cause of the formation of new or reinforcement of existing hydrogen bonds between water molecules. The aqueous medium is hostile to organic compounds, isolating them in clathrate-like cavities formed in an energetically favorable, exothermic process consisting in strengthening of hydrogen bonds among water molecules that form the hydration sheath [52]. Forming hydrogen bonds with its other molecules, water causes a geometric distribution of the hydrophobic interactions of organic molecules dissolved in it, causing the hydrophobic hydration to become a factor determining the increase in the $V_{\text{m}}^{^\circ }$ values of 15C5 within this concentration range of mixed solvent. In this process of solvent structure reconstruction, the exothermic enthalpic effect increases at the expense of decrease in entropy (entropy becomes more negative). Using literature data for the enthalpy of solution and enthalpy of sublimation or evaporation of 15C5 and urea in the mixtures of DMF + H_2_O [53–56], we calculated the solvation enthalpy (Δ_solv_ *H*°) of these compounds in the solvent mixtures of DMF and H_2_O at 298.15 K. The results are shown in Fig. 2. As is seen, the shape or course of the curve of solvation enthalphy is similar to the course of partial molar volume for 15C5. The increase of both values within the DMF-rich region (0 ≤ *x*_2_ ≈ 0.4) indicates difficulties in incorporating crown ether molecules into the mixed solvent structure. The increase in the exothermic enthalpic effect, above 0.92 water fraction, as it occurs in the process of crystallization, indicates an increase in the number of hydrogen bonds around the hydrophobic substance [51, 53]. This is the reason for the increasing values of $V_{\text{m}}^{^\circ }$ of 15C5.

Figure 1 shows a clear effect of temperature on the standard partial molar volumes of 15C5 within the whole mixture composition range. This value increases with increasing temperature as is expected. The increase in temperature weakens the interactions among molecules, through which the values of the standard partial molar volume of 15C5 are increased. In the system of DMF + H_2_O + urea, the course of the function $V_{\text{m}}^{^\circ }$ = *f*(*x*_2_) is different. As is seen in Fig. 1, the values of $V_{\text{m}}^{^\circ }$ for urea increase with increasing water content up to *x*_2_ ≈ 0.7, and then decrease [21]. Only within the area of *x*_2_ > 0.6 one can observe changes in the structure of mixed solvent brought about by the presence of urea molecules that form hydrogen bonds with water. Hydrophilic molecules of urea destroy the structure of the mixed solvent, within the water-rich region, which results in a decrease in the value of the standard partial molar volume of urea [21].

The linear temperature dependence of the partial molar volume of 15C5 allows calculation of the limiting apparent molar expansibilities (*α*) using Eq. 4:

4 $$ \alpha = \partial V_{m}^{^\circ } /\partial T $$

**Fig. 3:**
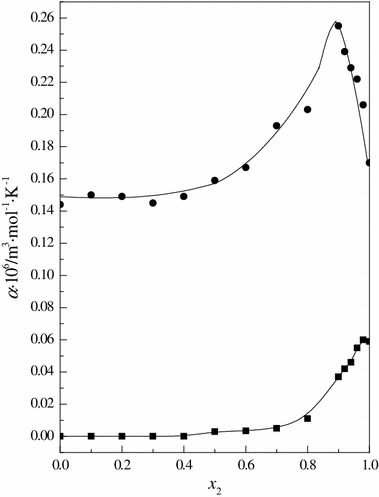
Limiting apparent molar expansibilities of *filled circle*, 15-crown-5, and *filled square*, urea in the solvent mixture (DMF + H_2_O)

The results are presented in Table 5 and Fig. 3. The values of limiting apparent molar expansibilities in water is in good agreement with literature data [57]. For comparison, in the same figure, data for the hydrophile urea are presented. These values were calculated using the data of the partial molar volumes of urea presented in our previous paper [21]. As in other cases, we can observe significant differences in the courses of the functions in the region *x*_2_ > 0.9. This reflects the differences in the hydrophobic and hydrophilic properties of molecules of 15C5 and urea.

**Table 5:** Limiting apparent molar expansibilities of 15C5 in the DMF + H_2_O mixtures

*x*_2_	*α* (cm^3^·mol^−1^·K^−1^)	*R* ^2^
0.00	0.144 ± 0.003	0.99936
0.10	0.150 ± 0.001	0.99990
0.20	0.149 ± 0.003	0.99870
0.30	0.145 ± 0.001	0.99998
0.40	0.149 ± 0.002	0.99978
0.50	0.159 ± 0.003	0.99942
0.60	0.167 ± 0.002	0.99976
0.70	0.193 ± 0.003	0.99956
0.80	0.203 ± 0.001	0.99994
0.90	0.255 ± 0.017	0.99170
0.92	0.239 ± 0.014	0.99331
0.94	0.229 ± 0.003	0.99960
0.96	0.222 ± 0.001	0.99992
0.98	0.206 ± 0.002	0.99972
1.00	0.170 ± 0.007 0.18^a^	0.99628

*x*_2_ is the mole fraction of water in the mixture

± is the standard deviation

*R* is the regression coefficient

^a^Bernal et al. [57]

In the low and medium water content mixtures no significant changes are observed in the course of the functions Δ_solv_ *H*° = *f*(*x*_2_), $V_{\text{m}}^{^\circ }$ = *f*(*x*_2_) and *α* = *f*(*x*_2_) for 15C5 and urea. Worthy of attention is the behavior of those functions in the mixtures with high water content. In this area the courses of all functions for 15C5 are completely opposite to that which we observed for urea. This is due to the differences in hydrophilic and hyrophobic properties of both investigated compounds. The exothermic process of hydrophobic hydration is the reason for increasing $V_{\text{m}}^{^\circ }$ values and decreasing of Δ_solv_ *H*° and *α*. In the water-rich region the interaction between the urea molecules weakens, and the interaction between them and water molecules becomes important. Completely reversed behavior of all functions for urea are caused by the destruction of the mixed solvent structure [21].

## Conclusion

The analysis of apparent (*V*_*Φ*_) and partial molar volumes ($V_{\text{m}}^{^\circ }$) of 15C5 in DMF + H_2_O mixtures made it possible to verify the changes in the mixed solvent structure occurring under the influence of dissolved hydrophobic 15C5. The analysis of the function $V_{\text{m}}^{^\circ }$ = *f*(*x*_2_) for 15C5 in DMF + H_2_O mixtures gives conclusions that the 15C5 molecules are hydrophobic hydrated at high water content of the mixture. The decreasing values of the virial coefficient *b*_V_ with increase of water content in the mixture indicate an increasing effect of hydrophobic hydration of 15C5 molecules in aqueous medium. The results for hydrophobic 15C5 were compared with analogous results for urea showing hydrophilic properties. The obtained conclusions concerning partial molar volume and expansibility of 15-crown-5 and urea in mixed solvent have been confirmed by the results of the solvation enthalpy of 15C5 and urea in the mixtures of DMF + H_2_O.
